# YAP and TEAD Are Transcriptional Regulators of Neuroendocrine Differentiation and Growth in Carcinoid Cells

**DOI:** 10.1016/j.ajpath.2025.10.012

**Published:** 2025-11-20

**Authors:** Jina Nanayakkara, Xiaojing Yang, Simona Damiani, Tashifa Imtiaz, Xiantao Wang, Dimitrios G. Anastasakis, Girish M. Shah, Kathrin Tyryshkin, Markus Hafner, Xiaolong Yang, Neil Renwick

**Affiliations:** ∗ Laboratory of Translational RNA Biology, Department of Pathology and Molecular Medicine, Queen's University, Kingston, Ontario, Canada; † RNA Molecular Biology Laboratory, National Institute of Arthritis and Musculoskeletal and Skin Diseases, Bethesda, Maryland; ‡ Centre Hospitalier Universitaire de Québec Université Laval, Faculty of Medicine, Department of Molecular Biology, Medical Biochemistry and Pathology, Laval University, Québec, Québec, Canada; § Cancer Research Laboratory, Department of Pathology and Molecular Medicine, Queen's University, Kingston, Ontario, Canada

## Abstract

Molecular regulators of variably aggressive carcinoid tumors are unknown. Since carcinoids have low expression of Yes-associated protein (YAP), it was hypothesized that low YAP expression provides a molecular advantage to carcinoids by preventing YAP from binding its partner, TEA domain transcription factor (TEAD). To test this hypothesis, constitutively active YAP and a TEAD-binding defective form of YAP were overexpressed in lung (H727) and pancreatic (BON1) carcinoid cells. It was found that active YAP overexpression inhibited neuroendocrine markers, morphology, cell proliferation, and anchorage-independent cell growth, whereas TEAD-binding defective YAP recovered these features. Through integrated chromatin immunoprecipitation and RNA sequencing analyses, it was found that YAP-TEAD binding down-regulated neuroendocrine transcription factor genes and up-regulated select transforming growth factor (TGF-β) superfamily and Notch genes related to cell growth. It was concluded that low YAP expression permits neuroendocrine differentiation and growth in carcinoid cells by preventing YAP-TEAD binding and subsequent dysregulation of gene targets. These results identify unknown molecular mechanisms in carcinoid development that may apply to the broader family of neuroendocrine cancers.

## Graphical abstract

**Figure:**
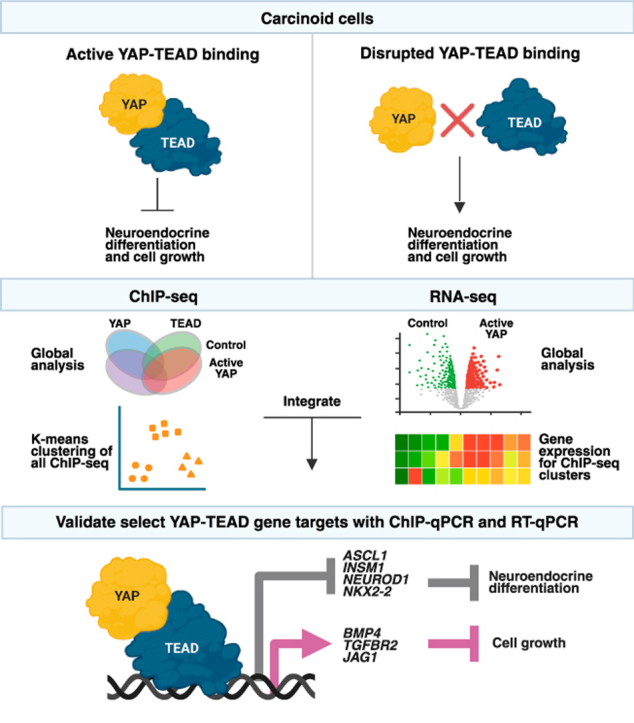

Carcinoids are well-differentiated, but variably aggressive, neuroendocrine tumors of the lung and gastroenteropancreatic tract.^1^^,^^2^ Despite their clinical diversity, carcinoids have common cellular and molecular features. Common cellular morphology includes a granular cytoplasm filled with dense neurosecretory vesicles, dendritic-like cell processes, clustered growth patterns, hormone secretion, and hormone receptors.^3^, ^4^, ^5^, ^6^ Carcinoids share molecular features like the expression of general neuroendocrine markers chromogranin A (CgA) and insulinoma-associated protein 1 (INSM1).^2^ Carcinoids also have a common transcription program^7^^,^^8^ with elevation of neuroendocrine transcription factors, like Achaete-scute family basic helix-loop-helix transcription factor 1 (ASCL1) and neuronal differentiation 1 (NEUROD1).^9^, ^10^, ^11^, ^12^ Although carcinoids share common features, molecular regulators of tumor development and growth are unknown.^1^ Understanding molecular regulators of variably aggressive carcinoids could provide insights into the biology of more aggressive neuroendocrine tumors or cancers.

Low Yes-associated protein (YAP) expression is a consistent molecular feature of carcinoids.^12^^,^^13^ YAP is a transcriptional cofactor in the Hippo signaling pathway, commonly known for its key role in regulating organ size, tissue homeostasis, regeneration, and tumorigenesis.^14^ YAP binds to cofactors, predominantly TEA domain transcription factors (TEAD),^15^^,^^16^ as well as many other transcription factors such as p73.^17^ The resultant YAP-TEAD complex binds DNA sequences at gene promoters or enhancers and regulates downstream gene expression.^16^ YAP-TEAD binding is critical for downstream gene expression because mutation^15^ or chemical inhibition^18^ of YAP-TEAD interaction sites prevents target gene expression. Many known YAP-TEAD target genes, such as *CTGF* and *CYR61*, drive tumorigenesis in non-neuroendocrine cancers.^19^^,^^20^ In contrast to non-neuroendocrine cancers,^20^^,^^21^ YAP is consistently lowly expressed in carcinoids.^12^^,^^13^ Further, YAP overexpression negatively regulates differentiation and growth in limited studies of normal neuroendocrine^22^, ^23^, ^24^ and carcinoid cells.^13^ Low YAP expression could be a critical molecular step permitting carcinoid development.^13^ Understanding the role and mechanism of YAP gene regulation in carcinoids is expected to provide insights into the molecular tumorigenesis of neuroendocrine cancers.

In this study, it was asked whether low YAP expression provides a molecular advantage for carcinoid differentiation and growth. Based on previous work,^13^ it was hypothesized that low YAP expression permits carcinoid development because high YAP expression allows YAP-TEAD binding. If so, highly expressed YAP-TEAD could negatively regulate neuroendocrine differentiation and growth through target gene dysregulation. To test this hypothesis, YAP-TEAD binding in the commonly used lung (H727) and pancreatic (BON1) carcinoid cells was disrupted using a TEAD-binding defective form of YAP^25^ or a potent chemical inhibitor of YAP-TEAD binding (VT104).^18^ Subsequently, YAP-TEAD DNA-binding sites were mapped using chromatin immunoprecipitation sequencing (ChIP-seq), measured target gene output with RNA sequencing (RNA-seq), and integrated ChIP-seq and RNA-seq to identify YAP-TEAD target genes. It was demonstrated that TEAD is the major effector of YAP in carcinoids and uncover YAP-TEAD gene targets relevant to carcinoid differentiation and growth.

## Materials and Methods

### Cell Lines and Cell Culture

Lung carcinoid (NCI-H727; H727) cells were obtained from the ATCC (Manassas, VA). Pancreatic carcinoid (BON1) and human embryonic kidney (HEK) 293T cells were obtained from Dr. Girish Shah (University of Laval) and Dr. Xiaolong Yang (Queen's University), respectively. H727 cells were cultured in ATCC-formulated RPMI-1640 (Invitrogen, Carlsbad, CA) medium supplemented with 10% fetal bovine essence. BON1 cells were cultured in Dulbecco’s modified Eagle’s medium/F12 1:1 (Invitrogen) supplemented with 10% fetal bovine essence and 1% penicillin/streptomycin (Invitrogen). HEK 293T cells were cultured in Dulbecco’s modified Eagle’s medium (Invitrogen) supplemented with 10% fetal bovine essence and 1% penicillin/streptomycin (Invitrogen). All lines were maintained at 37°C in a humidified 5% CO_2_ incubator. Brightfield microscopy images of cells were captured using the EVOS M7000 Imaging System (Thermo Fisher Scientific, Waltham, MA) and de-blurred using the Cellpose algorithm for cellular segmentation.^26^

### Plasmid Construction

Plasmids for doxycycline (Dox)-inducible constitutively active YAP (YAP-S127A)^27^ and TEAD-binding defective YAP (YAP-S127A/S94A)^25^ were generated as previously described.^13^^,^^28^ Briefly, the YAP-S127A/S94A insert was generated through overlapping PCR mutagenesis using the previously generated YAP-S127A plasmid as the template (Table 1). Active and TEAD-binding defective *YAP* genes lack the wild-type YAP 3′UTR.

**Table 1:** Primers for YAP-S127A/S94A Mutagenesis and Cloning into the pTRIPZ Vector, ChIP-qPCR, and RT-qPCR

Approach	Oligonucleotide	Sequence
Amplify YAP-S127A from pTRIPZ-YAP-S127A for subcloning	AgeI-YAP-F	5′-ATACCGGTACCATGGATCCCGGGCAGCAGCCG-3′
	MluI-YAP-R	5′-CGACGCGTCTATAACCATGTAAGAAAG-3′
Mutagenesis of YAP-S127A to YAP-S127A/S94A	YAP-S94A-Mut-F	5′-CGGAAGCTGCCCGACGCCTTCTTCAAGCCG-3′
	YAP-S94A-Mut-R	5′-CGGCGGCTTGAAGAAGGCGTCGGGCAGCTTCCG-3′
ChIP-qPCR	ASCL1-ChIP-F	5′-ATGGGGGTGAAAGAGTGCAT-3′
	ASCL1-ChIP-R	5′-ACTCCCAGTGACGCCTAGAT-3′
	BMP4-ChIP-F	5′-AAACTCCAGTGCTTCTCCACA-3′
	BMP4-ChIP-R	5′-TGTCAGCAGAAACCCTCGTAAA-3′
	INSM1-ChIP-F	5′-GGATTCGTGCAAACCTGGCT-3′
	INSM1-ChIP-R	5′-AGCCCACAAGCTTCAAGGTTTA-3′
	JAG1-ChIP-F	5′-ATGTTTGCTCTGCTGGTTCAGT-3′
	JAG1-ChIP-R	5′-GCTAGTGTGTTTAACCGATCCC-3′
	SMAD3-ChIP-F	5′-GGTACCCACGGGATGTCAGT-3′
	SMAD3-ChIP-R	5′-AGCTGGGCATCCTAGAAACA-3′
	SMAD6-ChIP-F	5′-GCAGGGGAGAAGGCCTATTG-3′
	SMAD6-ChIP-R	5′-CAATGTCCTGTGGCTGTCCT-3′
	TGFBR2-ChIP-F	5′-CAGCCTTGGTTAGAAAGCACAC-3′
	TGFBR2-ChIP-R	5′-AAGCGAGTTGCTTTTGCCTT-3′
RT-qPCR	ASCL1-F	5′-CGCTCGGCGGTCGAGTA-3′
	ASCL1-R	5′-GTTGTGCGATCACCCTGCTT-3′
	BMP4-F	5′-CTTCCACCACGAAGAACATCTG-3′
	BMP4-R	5′-ACCTCGTTCTCAGGGATGCT-3′
	INSM1-F	5′-TACGCGTTTGTCTCGTGGTT-3′
	INSM1-R	5′-CAGAGATTGGTAGGCGAGGC-3′
	JAG1-F	5′-CAGCCCTCATCCCTGTTACAA-3′
	JAG1-R	5′-AGGCACAAGGTGAAGACTGG-3′
	NEUROD1-F	5′-ATAGACCTGCTAGCCCCTCA-3′
	NEUROD1-R	5′-TGGTCATGTTTCGATTTCCTTTGTT-3′
	NKX2-2-F	5′-GTCCGGAGGAAGAGAACGAG-3′
	NKX2-2-R	5′-CCAGACCGTGCAGGGAGTA-3′
	SMAD3-F	5′-ACAGCATGGACGCAGGTTC-3′
	SMAD3-R	5′-CTCGCAGTAGGTAACTGGCT-3′
	SMAD6-F	5′-GGGCCCGAATCTCCGC-3′
	SMAD6-R	5′-ACATGCTGGCGTCTGAGAAT-3′
	TGFBR2-F	5′-CATTTGGTTCCAAGGTGCGG-3′
	TGFBR2-R	5′-CATCTGGATGCCCTGGTGG-3′

ChIP, chromatin immunoprecipitation; F, forward; qPCR, quantitative real-time PCR; R, reverse.

### Lentivirus Production and Cell Transduction

Lentiviruses for cell transduction were produced as previously described.^13^ Briefly, HEK 293T cells seeded in 100-mm plates were transfected with 2.5 μg of expression plasmid (pTRIPZ-YAP-S127A or pTRIPZ-YAP-S127A/S94A), 1.875 μg of packaging plasmid (psPAX2), and 0.626 μg of envelope plasmid (pMD2G) using PolyJet reagent (SignaGen Laboratories, Frederick, MD) according to the manufacturer's instructions. After transfection, HEK 293T cells were grown in Dulbecco’s modified Eagle’s medium (high glucose) containing 10% fetal bovine serum for 48 hours for recovery. Next, the lentivirus-containing medium was collected, filtered through a 0.45-μm cutoff (Sarstedt, Nümbrecht, Germany), and used for immediate transduction or flash frozen in liquid nitrogen and stored at −80°C.

H727 and BON1 cells were transduced with lentivirus to generate Dox-inducible active YAP overexpression (H727-YAP-S127A and BON1-YAP-S127A) or Dox-inducible TEAD-binding defective YAP overexpression (H727-YAP-S127A/S94A and BON1-YAP-S127A/S94A) cell lines. Briefly, cells were grown to 30% to 50% confluence in 6-well plates and medium was replaced with complete growth medium, lentivirus-containing medium, and 8 μg/mL Polybrene (Sigma-Aldrich, Burlington, MA) for 24 hours incubation at 37°C. Next, cells were re-infected with fresh lentivirus-containing medium for another 24 hours. Stable cell lines were selected with 2 μg/mL puromycin over 7 to 9 days. For experiments, stable cells were treated with 1 μg/mL doxycycline hyclate (BioShop Canada, Burlington, ON, Canada) for 48 to 96 hours before use.

### VT104

As another method of disrupting YAP-TEAD binding, H727 and BON1 were treated with a potent inhibitor of YAP-TEAD binding (VT104).^18^ This compound was purchased from AOBIOUS (AOB12135; AOBIOUS, Gloucester, MA).

### Western Blot Analysis

Cell lysates were collected and Western blots were performed as described.^13^ Primary antibodies included mouse monoclonal [C-12] anti-chromogranin A (sc393941, 1:500 dilution; Santa Cruz Biotechnology, Dallas, TX), mouse monoclonal [A-8] anti-INSM1 (sc271408, 1:200 dilution; Santa Cruz Biotechnology), mouse monoclonal [63.7] anti-YAP (sc-101199, 1:200 dilution; Santa Cruz Biotechnology), rabbit monoclonal anti–pan-TEAD (D3F7L, 1:1000 dilution; Cell Signaling Technology, Danvers, MA), and mouse monoclonal anti–β-actin antibody (A5441, 1: 6000 dilution; Sigma-Aldrich). Peroxidase-labeled secondary antibodies included anti-mouse IgG antibody (ab6789, 1:3000 dilution; Abcam, Cambridge, UK) or anti-rabbit IgG antibody (sc2357, 1:1000 dilution; Santa Cruz Biotechnology). If Western blots were probed for more than one primary antibody, blots were washed in TBS-T buffer for 30 minutes, blocked in 5% skim milk for 1 hour with gentle agitation, and washed again in TBS-T, before being reprobed with the second primary antibody.

### Cell Proliferation Assay

Cell proliferation assays were performed as previously described^13^^,^^29^ and repeated at least three times. For each cell line, 1.5 × 10^4^ cells were seeded into 12-well plates and counted daily for 6 days. To maintain YAP overexpression, culture medium containing DOX (1 μg/mL) was replaced on alternate days.

### Soft Agar Colony Formation Assay

Soft agar colony formation assays were performed as described^29^ and repeated at least three times. Briefly, 2 × 10^4^ cells (H727 transduced cell lines) or 1 × 10^4^ cells (BON1 transduced cell lines) were mixed with complete growth medium containing 0.4% agarose and overlaid on a 0.8% agarose layer in each well of a 6-well plate in triplicate. YAP overexpression was maintained with medium replacement (doxycycline 1 μg/mL) on alternate days as above. After incubation for 21 days (H727 transduced cell lines) or 18 days (BON1 transduced cell lines), colonies were stained with crystal violet (0.005% crystal violet in 20% methanol), imaged using a Bio-Rad Gel Doc System (Bio-Rad Laboratories, Hercules, CA), and counted using ImageJ software version 1.53 (NIH, Bethesda, MD; *https://imagej.net/ij*).

### ChIP Sequencing

YAP and TEAD ChIP sequencing was performed for active YAP overexpression and control cells in duplicate. First, protein–DNA interactions were reversibly crosslinked by treating cells with 1% formaldehyde (Thermo Fisher Scientific) for 10 minutes, and quenched with 125 mmol/L glycine (BioShop Canada) for 5 minutes. Cells were washed four times with cold phosphate-buffered saline and lysed in ChIP buffer [50 mmol/L Tris pH 7.5, 150 mmol/L NaCl, 5 mmol/L EDTA, 1% NP-40, 1% Triton X-100, 0.1% SDS, 1X Halt Protease Inhibitor Cocktail (Thermo Fisher Scientific)]. Chromatin was sheared with a Sonic Dismembrator Model 100 (Thermo Fisher Scientific) for 22 to 28 cycles (10 seconds on/30 seconds off). For post-sequencing data normalization, 1% of sheared chromatin input was retained and did not undergo the following immunoprecipitation.

Protein–DNA complexes were immunoprecipitated with 10 μg of rabbit monoclonal anti-YAP (14704, D8H1X; Cell Signaling Technology) or 10 μg of rabbit monoclonal anti-TEAD (13295, D3F7L; Cell Signaling Technology) overnight at 4°C with rotation. Antibody-chromatin complexes were isolated with ChIP-grade Protein G Magnetic Beads (Cell Signaling Technology) for 2 hours at 4°C with rotation. Following washes of the magnetic beads, protein–DNA crosslinking was reversed with 2-hour incubation at 65°C and proteinase K treatment (Cell Signaling Technology). Finally, DNA was extracted with QIAquick PCR purification kit (QIAGEN, Hilden, Germany) and quantitated by Qubit dsDNA high sensitivity assay (Thermo Fisher Scientific). Sequencing libraries were generated using a KAPA Hyperprep library kit (Roche Diagnostics, Pleasanton, CA), analyzed using a Bioanalyzer High Sensitivity DNA Chip, quantified by quantitative real-time PCR (qPCR), and sequenced (paired end) on an Illumina NovaSeq 6000 platform.

### ChIP-Seq Data Analysis

ChIP-seq output was quality controlled, aligned to the human genome, and analyzed with peak-calling tools. FASTQ sequence files were quality controlled using FastQC software version 0.11.9 (Babraham Bioinformatics, Cambridge, UK; *http://www.bioinformatics.babraham.ac.uk*/*projects/fastqc*) and adapter sequences and low-quality bases were trimmed using Trimmomatic software version 0.39.^30^ Quality-controlled sequencing reads were evaluated with the ENCODE Transcription Factor ChIP-seq 2 pipeline software version 2.2.0,^31^ as briefly described here. Reads were aligned to the human genome (hg38) using Bowtie2 software version 2.4.1^32^; uniquely mapped and properly paired reads were retained. PCR duplicate reads were removed using Picard Tools software version 2.23.3 (*http://broadinstitute.github.io/picard*). Peaks were called using MACS2 software version 2.2.7.^33^ A high-confidence peak set was selected using the IDR reproducibility framework^31^^,^^34^ (threshold: 0.05) to generate ChIP peak files. YAP and TEAD ChIP-seq reads were normalized to 1% of input chromatin sequencing reads and reported in ChIP signal files. Next, quality metrics were calculated for library complexity, peak reproducibility, signal-to-noise ratio, and peak enrichment. Peak and signal files were visualized using the UCSC genome browser^35^^,^^36^ and the Broad Institute Integrative Genome Viewer software version 2.15.1.^37^ ChIP peaks were grouped with k-means clustering using deepTools software version 3.5.0.^38^ Since k-means clustering requires user-defined *k*, k = 1 to k = 10 were tested; k = 6 was selected based on elbow plot analysis.

ChIP peaks were analyzed to identify *cis*-regulatory regions, transcription factor enrichment, biological pathways, and clustering patterns. Peaks were overlapped with ENCODE candidate *cis*-regulatory regions.^39^ Transcription factor enrichment analysis was performed using MEME-ChIP software version 5.5.0^40^ with the JASPAR 2022 database of transcription factor binding profiles.^41^ Peak-gene associations and pathway enrichment analyses were performed using GREAT software version 4.0.4^42^ with GO biological processes.^43^^,^^44^ A common set of peak coordinates was generated from all experimental conditions by merging overlapping peaks using BEDtools software version 2.30.0.^45^

### ChIP-qPCR

YAP or TEAD DNA-binding sites were validated using ChIP-qPCR. ChIP steps were similar to ChIP-seq above with the following modifications. Chromatin fragments were sheared for 15 to 20 cycles. IgG immunoprecipitation was added as a negative control; 2 μg of normal rabbit polyclonal anti-IgG (2729; Cell Signaling Technology). Magnetic beads were replaced with Protein A/G agarose beads (sc2003; Santa Cruz Biotechnology). De-crosslinking consisted of boiling for 10 minutes and purified DNA was used immediately for ChIP-qPCR.

ChIP-qPCR reactions included 1 × PowerUp SYBR Green PCR Master Mix (Thermo Fisher Scientific), purified ChIP DNA, and 500 nmol/L peak specific primers (Table 1). Relative YAP or TEAD DNA-binding was quantitated on a ViiA 7 Real-Time PCR System (Thermo Fisher Scientific) using the following thermal cycling conditions: 50°C for 2 minutes, 95°C for 2 minutes, 40 cycles of 95°C for 15 seconds, and 60°C for 1 minute. YAP or TEAD DNA-binding signal was calculated as a percentage of input chromatin. Experiments were performed in technical duplicates and repeated at least three times.

### RNA Isolation and Quality Control

Total RNA was isolated from active YAP overexpression and TEAD-binding defective YAP overexpression cells using TRIzol Reagent (Invitrogen) according to the manufacturer's instructions. Total RNA was quality controlled through UV spectrophotometry using a SmartSpec Plus Spectrophotometer (Bio-Rad Laboratories) and visualization through 1% agarose gel electrophoresis.

### RNA-Sequencing and Annotation

RNA-seq was performed in triplicate for control and active YAP overexpression cells as described.^13^ RNA-seq data from the H727 cell line was previously published.^13^ Briefly, cDNA libraries were prepared using the NEBNext Ultra RNA Library Prep Kit (NEB E7530; New England Biolabs, Ipswich, MA) and the NEBNext rRNA Depletion Kit, NEB E6310; New England Biolabs) according to the manufacturer's instructions; RNA fragmentation was performed for 10 minutes. Libraries were sequenced on an Illumina NovaSeq 6000 platform (paired end). Raw sequence reads were de-multiplexed, quality controlled using FastQC software version 0.11.9, trimmed with Trimmomatic software version 0.39, and aligned to the human reference transcriptome GRCh38 (release 97) using Kallisto software version 0.46.1. Lowly expressed genes were filtered by 80th percentile expression in at least one sample. Differentially expressed genes were identified by 1.5-fold change in median gene expression (transcripts per million) between control and active YAP overexpression cells. Pathway enrichment analyses were performed on differentially expressed genes using gProfiler.^46^

### Quantitative Real-Time PCR

Predicted YAP and TEAD target genes were quantified using qPCR as described.^13^ Following bulk first-strand cDNA synthesis using SuperScript IV VILO First-Strand Synthesis System (Thermo Fisher Scientific), mRNAs of interest and 18S rRNA were quantitated on a ViiA 7 Real-Time PCR System (Thermo Fisher Scientific). Each reaction comprised cDNA from 25 ng of initial RNA input, 2X PowerUp SYBR Green Master Mix (Thermo Fisher Scientific), and 500 nmol/L gene-specific primers (Table 1). PCR amplification used the following thermal cycling conditions: 50°C for 2 minutes, 95°C for 2 minutes followed by 40 cycles of 95°C for 15 seconds and 60°C for 1 minute. Mean and SEM threshold cycle (C_T_) values for each target were obtained and relative mRNA expression calculated using the 2^−ΔΔC_T_^ method.^47^

### Integration of ChIP-Seq and RNA-Seq Data

ChIP-seq and RNA-seq data were integrated by defining potential peak-gene regulatory relationships using GREAT. GREAT defines a gene regulatory domain as a “basal domain that extends 5 kb upstream and 1 kb downstream from its transcription start site, and an extension up to the basal regulatory domain of the nearest upstream and downstream genes within 1 Mb.”^42^^,p.495^ For any ChIP peak within a gene regulatory domain, it was considered to be a peak-gene relationship. Gene expression was extracted from RNA-seq for genes with peak-gene relationships. Since multiple ChIP peaks could be associated with the same gene, one gene could be associated with multiple ChIP peak clusters.

### Statistical Analyses

Two-sided *t*-test was used to evaluate significant differences (*P* < 0.05) between groups. *P* values are indicated in the figures as: ∗*P* < 0.05, ∗∗*P* < 0.01.

### Data Availability

ChIP-sequencing and RNA-sequencing data were deposited in Gene Expression Omnibus (GEO) GSE301506 (*https://www.ncbi.nlm.nih.gov/geo*; accession number GSE301506) and GSE301503 (*https://www.ncbi.nlm.nih.gov/geo*; accession number GSE301503), respectively. RNA-seq data from the H727 cell line was previously published.^13^

## Results

### YAP-TEAD Binding Controls Neuroendocrine Differentiation in Carcinoid Cells

**Figure 1:**
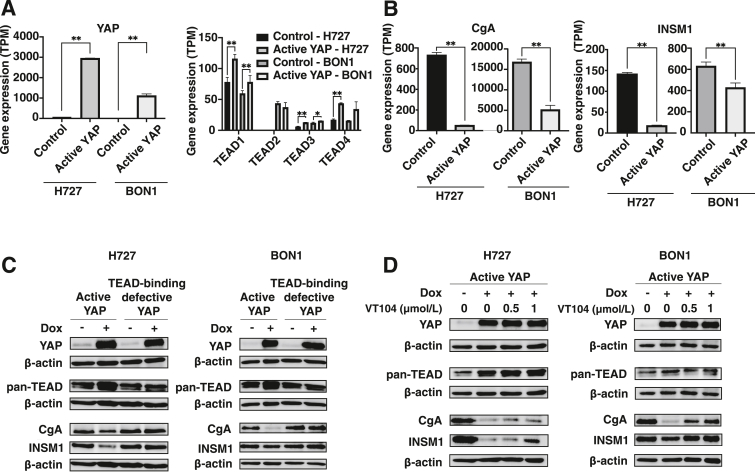
Disrupted YAP-TEAD binding inhibits neuroendocrine differentiation in carcinoid cells. Baseline gene expression of YAP and TEAD (**A**) and CgA and INSM1 (**B**). Gene expression was extracted from RNA-seq for control or active YAP overexpression cells. Western blots of CgA and INSM1 in response to disrupted YAP-TEAD binding with TEAD-binding defective YAP (**C**) or VT-104 (**D**). Active or TEAD-binding defective YAP was overexpressed with 72- to 96-hour doxycycline (Dox) induction. Active YAP overexpression cells were treated with the YAP-TEAD binding inhibitor (VT104) for 72 hours. Data are expressed as means ± SEM. *n* = 3 control cells; *n* = 3 active YAP cells. ∗*P* < 0.05, ∗∗*P* < 0.01 by *t*-test. TPM, transcripts per million.

To better understand the link between low YAP expression and neuroendocrine differentiation in carcinoid tumors,^12^^,^^13^ constitutively active YAP (YAP-S127A)^27^ and TEAD-binding defective YAP (YAP-S127A/S94A)^25^ were overexpressed in lung (H727) and pancreatic (BON1) carcinoid cell lines, and changes in neuroendocrine marker expression (CgA and INSM1) and cell morphology were compared. Low levels of endogenous YAP mRNA and abundant TEAD isoform mRNAs were detected in both control cell lines (Figure 1A). When active YAP was overexpressed, CgA and INSM1 mRNA and protein levels were reduced (Figure 1, B and C). When TEAD-binding defective YAP was overexpressed, CgA and INSM1 protein levels increased relative to active YAP overexpression (Figure 1C). After disrupting YAP-TEAD binding through VT-104 chemical inhibition, dose-dependent increases in CgA and INSM1 protein expression were similarly observed (Figure 1D). Morphologic changes were also observed after disrupting YAP-TEAD binding. When active YAP was overexpressed, cells changed shape to be more adherent (Supplemental Figure S1). When TEAD-binding defective YAP was overexpressed, cells reacquired their original cell shape (Supplemental Figure S1). Taken together, active YAP-TEAD binding is associated with decreased neuroendocrine marker expression and loss of neuroendocrine morphology, indicating that YAP-TEAD binding inhibits neuroendocrine differentiation in H727 and BON1 cell lines.

### YAP-TEAD Binding Represses Cell Proliferation and Growth in Carcinoid Cells

**Figure 2:**
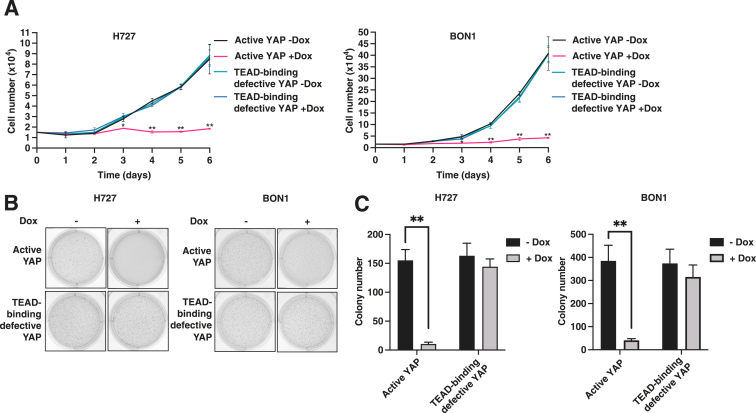
Disrupted YAP-TEAD binding reduces cell proliferation and growth in carcinoid cells. **A:** Cell proliferation. Cells were seeded at 1.5 × 10^4^ and counted daily for control (−Dox) and active or TEAD-binding defective YAP overexpression (+Dox) conditions. **B** and **C**: Soft agar assay. Cells were seeded in soft agar and assessed for colony formation for control (−Dox) and active or TEAD-binding defective YAP overexpression (+Dox) conditions. Data are expressed as means ± SEM. *n* = 3 −Dox (**A**); *n* = 3 +Dox (**A**); *n* = 7 to 9 −Dox (**B** and **C**); *n* = 9 +Dox (**B** and **C**). ∗*P* < 0.05, ∗∗*P* < 0.01 by *t*-test. Dox, doxycycline.

To understand the relationship between low YAP expression and cell growth in carcinoid tumors,^13^ cell proliferation and soft agar colony formation assays were performed on active YAP and TEAD-binding defective YAP overexpression conditions in H727 and BON1 cells. Cell proliferation was significantly reduced in both cell lines following active YAP, but not TEAD-binding defective YAP, overexpression (Figure 2A). Similarly, anchorage-independent growth in the soft agar assay significantly decreased after active YAP, but not TEAD-binding defective YAP, overexpression (Figure 2, B and C). Taken together, active YAP-TEAD binding was associated with a significant reduction in cell proliferation and anchorage-independent growth, indicating that YAP-TEAD binding represses cell growth in H727 and BON1 cells.

### Global YAP and TEAD DNA-Binding Patterns Are Nonspecific in Carcinoid Cells

**Figure 3:**
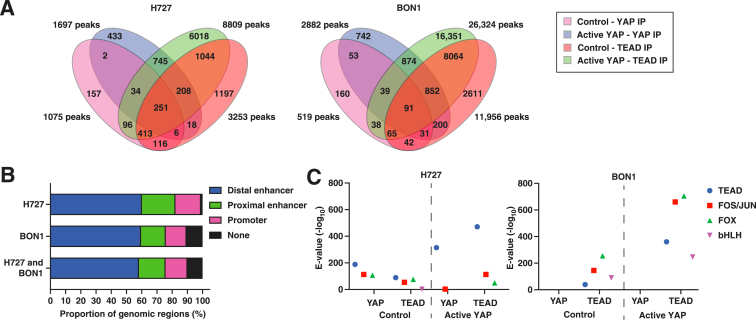
Global YAP and TEAD DNA-binding patterns in carcinoid cells. **A:** Venn diagram of YAP and TEAD chromatin immunoprecipitation (ChIP) peaks. **B:***Cis*-regulatory region. ChIP peaks were intersected with candidate *cis*-regulatory regions from ENCODE. **C:** Motif enrichment. Overrepresented transcription factor motifs were detected by MEME-ChIP and ranked by significance (e-value <0.05) (Supplemental Table S2). Basic helix-loop-helix (bHLH) transcription factors included ATOH7, BHLHA15, NEUROD2, HAND2, TAL1, HES6, and ASCL1. YAP peaks in BON1 were only enriched in low complexity sequence motifs (not shown). IP, immunoprecipitation.

To gain insights into how YAP-TEAD binding represses neuroendocrine cell differentiation and growth in H727 and BON1 cells, YAP and TEAD DNA-binding sites were mapped using ChIP-seq. Unique and overlapping YAP and TEAD ChIP peaks were found for and between control and active YAP overexpression cells (Figure 3A and Supplemental Tables S1 and S2). The majority of ChIP peaks (58%) were located in distal enhancers predicted by the ENCODE project^39^; other ChIP peaks were located in proximal enhancers (17%), promoters (15%), or outside known regulatory regions (9%) (Figure 3B). Based on MEME-ChIP analysis of ChIP peaks, there was significant transcription factor enrichment for TEAD, FOS/JUN, FOX, and basic helix-loop-helix (bHLH) motifs (Figure 3C and Supplemental Table S3). Using GREAT pathway analysis for ChIP peaks, YAP and TEAD DNA-binding sites were enriched near genes involved in a wide range of biological functions from chromatin silencing to EGFR activity (Supplemental Table S4). Based on these global analyses, YAP and TEAD predominantly bind to distal enhancers and potentially interact with transcription cofactors. However, specific biological functions relevant to carcinoids were not identified.

### Clustered YAP and TEAD DNA-Binding Patterns Have Specific Biological Functions in Carcinoid Cells

**Figure 4:**
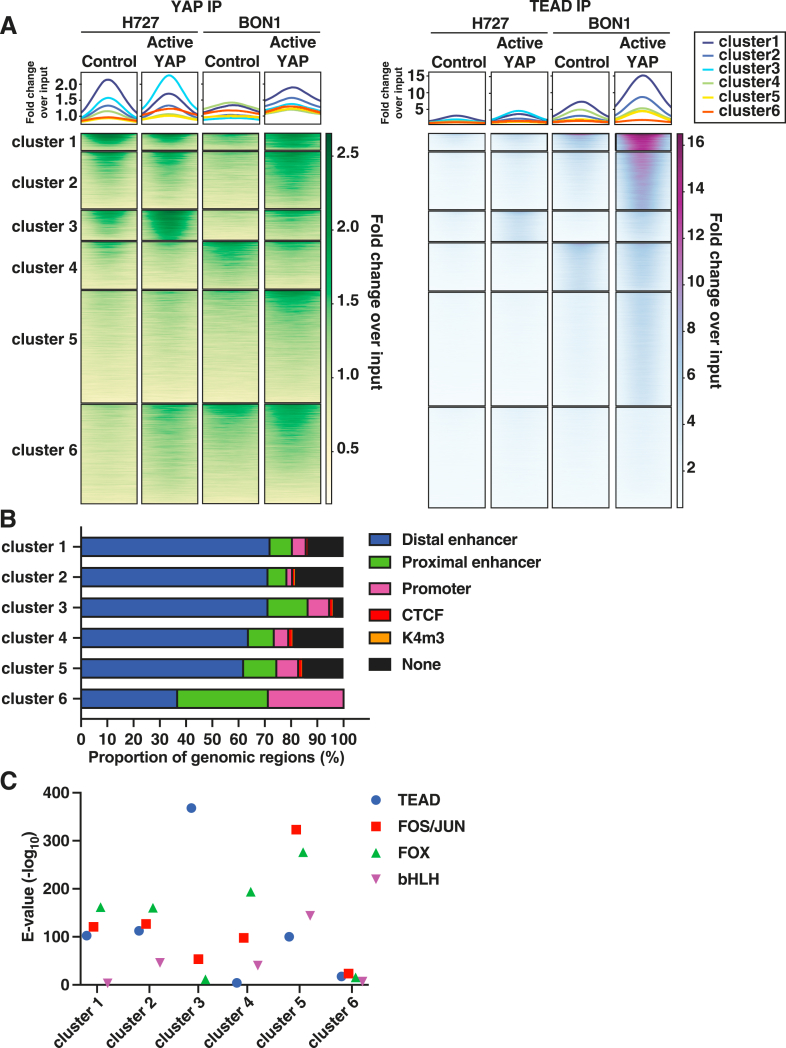
Clustered YAP and TEAD DNA-binding patterns in carcinoid cells. **A:** Heatmaps of YAP and TEAD chromatin immunoprecipitation sequencing (ChIP-seq). ChIP-seq signal represents fold change of YAP or TEAD immunoprecipitation (IP) sequencing reads relative to input chromatin. Cluster-specific heatmaps are presented in Supplemental Figure S2. **B:***Cis-*regulatory region. Cluster-specific ChIP peaks were intersected with candidate *cis-*regulatory elements from ENCODE. CTCF and K4m3 represent CTCF DNA-binding sites or K4m3 histone marks not otherwise classified as promoter or enhancer regions. **C:** Motif enrichment. For cluster-specific ChIP peaks, enriched transcription factor motifs were detected by MEME-ChIP and ranked by significance (e-value <0.05) (Supplemental Table S2). Basic helix-loop-helix (bHLH) transcription factors included NEUROD1, NEUROD2, NEUROG2, ATOH1, BHLHA15, TAL1, HAND2, OLIG2, TWIST2, and HES1. *n* = 2 YAP IP sequencing reads; *n* = 2 TEAD IP sequencing reads.

To further explore the YAP and TEAD ChIP-seq data in H727 and BON1 cells, k-means clustering analysis was performed and six predicted clusters of ChIP peaks were identified in both cell lines (Figure 4A and Supplemental Table S1). Similar to the global analyses above, most cluster-specific ChIP peaks were located in distal enhancers (Figure 4B). Cluster-specific ChIP peaks were significantly enriched in TEAD, FOS/JUN, FOX, and bHLH motifs (Figure 4C and Supplemental Table S2). Using GREAT pathway analysis, distinct biological functions were found for four predicted clusters including: chromatin remodeling (cluster 1), MAPK signaling (cluster 2), cardiovascular and bone development including TGF-β and Notch signaling (cluster 3), and neuron development and endocrine pancreas (cluster 4); overly diverse or nonspecific biological functions were found for clusters 5 and 6 (Supplemental Table S3). From these exploratory analyses, it was found that YAP and TEAD DNA-binding potentially regulates gene targets involved in several distinct biological functions.

### YAP and TEAD DNA-Binding Is Associated with Global and Cluster-Specific Gene Expression Patterns in Carcinoid Cells

**Figure 5:**
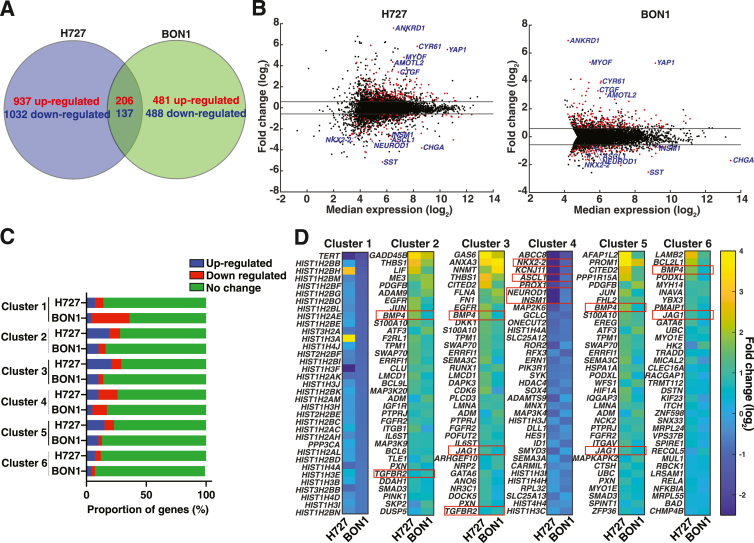
YAP-TEAD regulate global and clustered gene expression patterns in carcinoid cells. **A:** Venn diagram of dysregulated genes. Dysregulated genes were identified by a 1.5-fold threshold comparing active YAP overexpression to control. **B:** Volcano plots. Shared dysregulated genes between H727 and BON1 are highlighted in red; including known YAP target genes (*ANKRD1, CTGF, CYR61, AMOTL2,*and *AJUBA*), neuroendocrine transcription factors (*ASCL1, NEUROD1, NKX2-2,* and *INSM1*). and other neuroendocrine-related genes (*CHGA* and *SST*). **C:** Dysregulated genes per chromatin immunoprecipitation (ChIP) peak cluster. **D:** Gene expression heatmaps for genes associated with ChIP peak clusters. The top 35 up-regulated or down-regulated genes associated with ChIP peaks (Supplemental Table S6) are presented based on patterns in **C**. Fold change (log_2_) represents gene expression changes comparing active YAP overexpression to control. Neuroendocrine transcription factors, TGF-β and Notch genes are highlighted in **red boxes**. Since multiple ChIP peaks could be associated with the same gene, one gene could be associated with multiple ChIP peak clusters. TPM, transcripts per million.

To identify gene expression patterns associated with YAP and TEAD DNA-binding in H727 and BON1 cells, RNA-seq was performed on control and active YAP overexpression cells and integrated RNA-seq with ChIP-seq data. The authors previously reported dysregulated genes following active YAP overexpression in H727^13^ and currently report results in BON1 (Supplemental Table S4). Through global analysis, a common set of 206 up-regulated and 137 down-regulated genes following active YAP overexpression were found (Supplemental Table S5 and Figure 5A). The top up-regulated genes included *ANKRD1*, *YAP*, and *CYR61*; *ANKRD1* and *CYR61* are known YAP-TEAD gene targets (Figure 5B).^20^ The top down-regulated genes included *SST*, *LCN15*, and *CHGA*; *SST* and *CHGA* are neuroendocrine-related genes. Up-regulated pathways had common themes of cell adhesion and extracellular matrix components. Down-regulated pathways were related to neuroendocrine differentiation. For genes associated with ChIP-seq clusters from the previous section, active YAP overexpression was found to be associated with up-regulation (range: 5% to 33%), down-regulation (range: 5% to 38%), or no change in gene expression (range: 53% to 87%) (Figure 5C). Cluster 1 genes (histone-related) were frequently down-regulated (Figure 5D and Supplemental Table S6). Clusters 2 and 3, including TGF-β and Notch genes, were up-regulated. Cluster 4 genes (neuroendocrine transcription factors) were down-regulated. Cluster 5 and 6 also included upregulated TGF-β and Notch genes. After integrating RNA-seq with ChIP-seq data, YAP and TEAD DNA-binding was associated with global and cluster-specific gene dysregulation.

### YAP-TEAD Binds Proximal Genomic Regulatory Regions to Putatively Activate Select TGF-β and Notch Genes Related to Cell Growth in Carcinoid Cells

**Figure 6:**
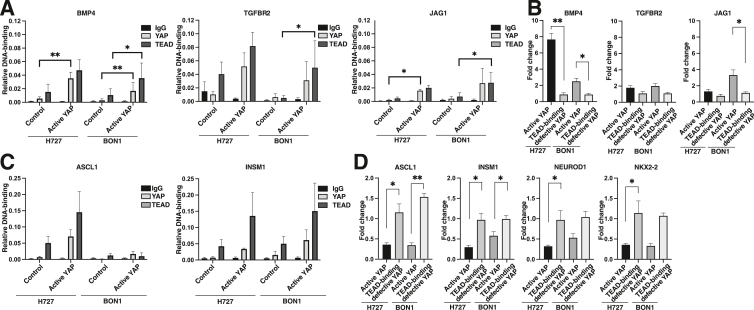
Chromatin immunoprecipitation quantitative PCR (ChIP-qPCR) and RT-qPCR validation of putative YAP-TEAD target genes in carcinoid cells. **A:** ChIP-qPCR validation of TGF-β and Notch genes. ChIP-qPCR for YAP and TEAD peaks in promoters or proximal enhancers of *BMP4, TGFBR2,* and *JAG1* genes. All C_T_ values were normalized to input chromatin. **B:** RT-qPCR validation of TGF-β and Notch genes. **C:** ChIP-qPCR validation of neuroendocrine transcription factors. ChIP-qPCR was performed for select YAP and TEAD peaks in distal enhancers surrounding *ASCL1* and *INSM1* genes. All C_T_ values were normalized to input chromatin. **D:** RT-qPCR validation of neuroendocrine transcription factors. Data are expressed as means ± SEM. ∗*P* < 0.05, ∗∗*P* < 0.01 by *t* test.

To understand the relationship between active YAP-TEAD and up-regulated *BMP4*, *TGFBR2*, and *JAG1* genes, this study focused on YAP-TEAD DNA-binding and gene expression patterns (Figure 5D). TGF-β and Notch signaling have been linked to decreased growth of neuroendocrine cells.^22^^,^^48^ When active YAP was overexpressed, ChIP peaks from cluster 3 had increased YAP and TEAD DNA-binding (Figure 4 and Supplemental Figure S2). Some of these ChIP peaks were found within promoters or proximal enhancers of *BMP4* (TGF-β superfamily), *TGFBR2* (TGF-β receptor), and *JAG1* (Notch ligand). When active YAP was overexpressed, higher YAP and TEAD DNA-binding was confirmed for select ChIP peaks in promoters or proximal enhancers of these BMP4/TGF-β and Notch genes with ChIP-qPCR (Figure 6A). Similarly, increased gene expression of *BMP4*, *TGFBR2*, and *JAG1* after active YAP, but not TEAD-binding defective YAP, overexpression was found (Figure 6B). Taken together, active YAP-TEAD is associated with increased YAP-TEAD DNA-binding at promoters and proximal enhancers up-regulating *BMP4*, *TGFBR2*, and *JAG1* genes.

### YAP-TEAD Binds Distal Genomic Regulatory Regions and Putatively Represses Neuroendocrine Transcription Factor Expression in Carcinoid Cells

To understand the relationship between active YAP-TEAD and down-regulated neuroendocrine transcription factor genes, the study focused on DNA-binding and gene expression patterns in cluster 4. When active YAP was overexpressed, ChIP peaks from cluster 4 had decreased YAP and unchanged TEAD DNA-binding (Figure 4 and Supplemental Figure S2). These ChIP peaks were found within predicted distal enhancers surrounding the neuroendocrine transcription factors *ASCL1*, *INSM1*, *NEUROD1*, and *NKX2-2* (Supplemental Figure S3). YAP and TEAD DNA-binding for select ChIP peaks surrounding *ASCL1* and *INSM1* were validated with ChIP-qPCR (Figure 6C). Next, decreased gene expression of *ASCL1*, *INSM1*, *NEUROD1,* and *NKX2-2* after active YAP, but not TEAD-binding defective YAP, overexpression were detected (Figure 6D). Taken together, active YAP-TEAD is associated with YAP-TEAD DNA-binding at distal enhancers down-regulating neuroendocrine transcription factor genes.

## Discussion

Although molecular regulators of variably aggressive carcinoids are unknown, low YAP expression is a consistent molecular feature of carcinoids.^12^^,^^13^ In this study, it was asked whether low YAP expression provides a molecular advantage for lung and pancreatic carcinoid cells. Highly expressed YAP was found to bind to TEAD to inhibit neuroendocrine differentiation and cell growth in carcinoid cells. Active YAP-TEAD binding inhibited neuroendocrine markers and cell morphology, but disrupted YAP-TEAD binding restored neuroendocrine features. Similarly, active YAP-TEAD binding repressed cell proliferation and anchorage-independent cell growth, but disrupted YAP-TEAD binding restored cell growth. This demonstrates that TEAD is the major effector of YAP-mediated neuroendocrine and growth repression in carcinoid cells. This builds on previous work that YAP-TEAD binding inhibits neuroendocrine differentiation and growth in normal lung and pancreatic progenitor cells^22^, ^23^, ^24^ and high-grade neuroendocrine carcinomas.^49^^,^^50^ Surprisingly, YAP-TEAD is oncogenic in multiple non-neuroendocrine solid tumor types,^16^^,^^21^ but this study and others^49^^,^^50^ reveal YAP-mediated differentiation and growth repression in a neuroendocrine cell-type–specific context. Through the authors’ experiments, low YAP expression was shown to provide a molecular advantage to carcinoids by preventing YAP-TEAD inhibition of neuroendocrine differentiation and cell growth.

After establishing that YAP-TEAD binding inhibits neuroendocrine differentiation in carcinoid cells, it was asked how YAP-TEAD exert this function. Using integrated ChIP-seq and RNA-seq, YAP-TEAD was shown to putatively target and down-regulate the neuroendocrine transcription factors *ASCL1*, *NEUROD1*, *INSM1,* and *NKX2-2*. As evidence, YAP-TEAD DNA-binding sites were found in distal enhancers surrounding these genes. Active YAP-TEAD binding down-regulated expression of these gene targets, but disrupted YAP-TEAD binding recovered gene expression. Previously unknown gene targets of YAP-TEAD relevant to neuroendocrine differentiation were elucidated in carcinoid cells. The finding of YAP-TEAD down-regulation of neuroendocrine transcription factors aligns with inverse gene expression patterns between YAP and neuroendocrine transcription factors in multiple neuroendocrine cell types.^13^^,^^22^^,^^51^ ASCL1,^52^, ^53^, ^54^ NEUROD1,^54^ and INSM1^55^^,^^56^ can drive neuroendocrine transdifferentiation from non-neuroendocrine lung or pancreatic cells. INSM1 is necessary for neuroendocrine cell development in the lung^57^ and gastroenteropancreatic tract.^58^ NKX2-2 is required for terminal differentiation of pancreatic β-cells.^59^ These findings demonstrate that YAP-TEAD binding inhibits neuroendocrine differentiation in carcinoid cells through putative down-regulation of neuroendocrine transcription factors.

Next, it was asked how YAP-TEAD binding inhibits growth in carcinoid cells. YAP-TEAD were shown to regulate putative gene targets including *BMP4*, *TGFBR2*, and *JAG1* genes. As evidence, YAP-TEAD DNA-binding sites were detected in promoters or proximal enhancers of these genes. Active YAP-TEAD binding up-regulated expression of these gene targets, but disrupted YAP-TEAD binding recovered gene expression. Unknown gene targets of YAP-TEAD relevant to cell growth in carcinoid cells were previously elucidated. The identification of YAP-TEAD up-regulation of *BMP4* and *TGFBR2* is consistent with activated TGF-β signaling inhibiting BON1 cell growth in previous studies.^60^ Similarly, the study’s finding of YAP-TEAD up-regulation of *JAG1* is consistent with YAP-TEAD activation of Notch signalling^22^^,^^51^ to inhibit cell growth in lung neuroendocrine carcinomas. Taken together, YAP-TEAD inhibits carcinoid cell growth through putative up-regulation of *BMP4*, *TGFBR2*, and *JAG1* genes.

Through ChIP-seq and RNA-seq, patterns of YAP-TEAD DNA-binding and gene dysregulation were identified. This study’s findings match discoveries that 91% to 95% of YAP-TEAD DNA-binding is within distal enhancers.^23^^,^^61^ Consistent with previous work, AP-1^61^ (FOS/JUN) and bHLH^49^ were identified as potential transcription co-regulators with YAP-TEAD. Although most bHLH transcription factors were minimally expressed in control carcinoid cells, NEUROD1 was highly expressed, indicating that YAP-TEAD and NEUROD1 could regulate the same gene targets in different contexts. Through k-means clustering analysis, ChIP-seq clusters were found that could represent diverse biological functions of YAP,^62^ or differential YAP-TEAD DNA-binding between lowly expressed endogenous YAP and overexpressed active YAP. From RNA-seq, the top up-regulated and down-regulated genes matched known YAP-TEAD target genes^20^ or neuroendocrine-related genes such as *SST*,^1^^,^^2^^,^^63^ respectively. Interestingly, SST functions as an autocrine negative regulator of hormone secretion and cell proliferation through somatostatin receptors (SSTRs).^64^ Active YAP-TEAD down-regulation of both *SST* and *SSTR5* could explain why decreased SST did not increase expression of hormone or proliferation-related genes. YAP-TEAD targeting of cell adhesion and extracellular matrix genes were also detected, consistent with recent studies^49^ and the well-known role of YAP in mechanotransduction.^24^ Mapping YAP-TEAD DNA-binding sites and measuring target gene transcription detected consistent and new patterns for further study in carcinoids.

This study of YAP-TEAD binding and gene regulation in carcinoid cells provides opportunity for further study. Although models of carcinoids are lacking,^6^ two carcinoid cell lines were used that have not previously been used to study YAP-TEAD binding. Further studies could demonstrate direct evidence of YAP-TEAD binding and inhibition of YAP-TEAD binding with VT104 through co-IP in carcinoid cells. Here, it was discovered YAP-TEAD distal gene regulation using candidate *cis*-regulatory elements derived from many cell lines.^39^ Since *cis*-regulatory elements may be cell-type specific, future investigations should define *cis-*regulatory elements in carcinoid cells through ChIP-seq for histone modifications or ATAC-seq for chromatin accessibility.^65^ Although largescale validation of enhancer-gene interactions remains challenging,^65^ further studies could genetically manipulate YAP-TEAD DNA-binding sites to validate gene targets. Finally, these findings of carcinoid specific gene targets of YAP-TEAD should be evaluated in other well-differentiated neuroendocrine tumors as more RNA-sequencing data become available. Despite these limitations, this study provides new evidence of YAP-TEAD regulation of neuroendocrine differentiation and growth.

In summary, it was discovered that YAP-TEAD inhibits carcinoid differentiation and cell growth through dysregulation of specific gene targets. This identifies new molecular mechanisms underlying carcinoid development. The study demonstrates that TEAD is the major effector of YAP-mediated repression of neuroendocrine differentiation and cell growth. and proposes that select TGF-β, Notch, and neuroendocrine transcription factor genes are important YAP-TEAD gene targets in carcinoid cells. Although YAP is oncogenic in non-neuroendocrine cancers, these findings are surprising because YAP-TEAD inhibited carcinoid cell growth. Stable YAP expression could be applied as a therapeutic strategy in carcinoids. Although YAP overexpression could have unintended effects, inhibiting miR-375, an inhibitor of YAP, could be a therapeutic strategy resulting in moderate, stable YAP expression.^13^ The function of YAP-TEAD and its gene targets in carcinoid cells may be generalizable to other neuroendocrine tumors, neuroendocrine carcinomas, or cancers that undergo neuroendocrine transdifferentiation for therapeutic resistance. Stable YAP expression through miR-375 inhibition may be a therapeutic strategy for these cancers, or even an adjunct therapy that prevents neuroendocrine transdifferentiation. This study of YAP-TEAD and carcinoid-specific gene targets contributes new insights into molecular mechanisms of carcinoid development with implications to the broader family of neuroendocrine cancers.

## Disclosure Statement

None declared.

## Supplemental Data

Supplemental Table S6

Supplemental Figure S1 Disrupted YAP-TEAD binding inhibits neuroendocrine morphology in carcinoid cells. Bright-field microscopy at ×200 total magnification for control (−Dox), active YAP, or TEAD-binding defective YAP overexpression (+Dox) cells. When active YAP was overexpressed in H727, cells lost their thin dendritic-like cell processes (**arrows**). Similarly, BON1 cells lost their rounded shape (**arrows**). Cell images were deblurred using the Cellpose deblurring algorithm and presented alongside original images. Scale bar: 100 μm. Original magnification: ×200. Dox, doxycycline.

Supplemental Figure S2 YAP and TEAD chromatin immunoprecipitation sequencing (ChIP-seq) based on k-means clustering. Cluster-specific heatmaps. ChIP-seq signal represents fold change of YAP (**A**) or TEAD (**B**) immunoprecipitation (IP) sequencing reads relative to input chromatin. *n* = 2 YAP sequencing reads; *n* = 2 TEAD sequencing reads.

Supplemental Figure S3 Integrative genome view of ASCL1, INSM1, NEUROD1, and NKX2-2. Chromatin immunoprecipitation (ChIP) peaks (YAP and TEAD DNA-binding sites) were identified within promoters and proximal enhancers of *BMP4, TGFBR2,* and *JAG1* or within 1 Mb of the transcription start site (TSS) of *ASCL1*, *INSM1*, *NEUROD1,* and *NKX2-2*. Predicted promoter and enhancer regions were obtained from ENCODE. ChIP-seq tracks were displayed for control and active YAP overexpression conditions in H727 and BON1, with YAP or TEAD immunoprecipitation (IP).
