# Supplementary figures and images for: YAP and TEAD Are Transcriptional Regulators of Neuroendocrine Differentiation and Growth in Carcinoid Cells
Source: Am J Pathol. 2025 Nov 20;196(2):345–58. doi: 10.1016/j.ajpath.2025.10.012 (PMC12881680; doi:10.1016/j.ajpath.2025.10.012)

### De-blurred images

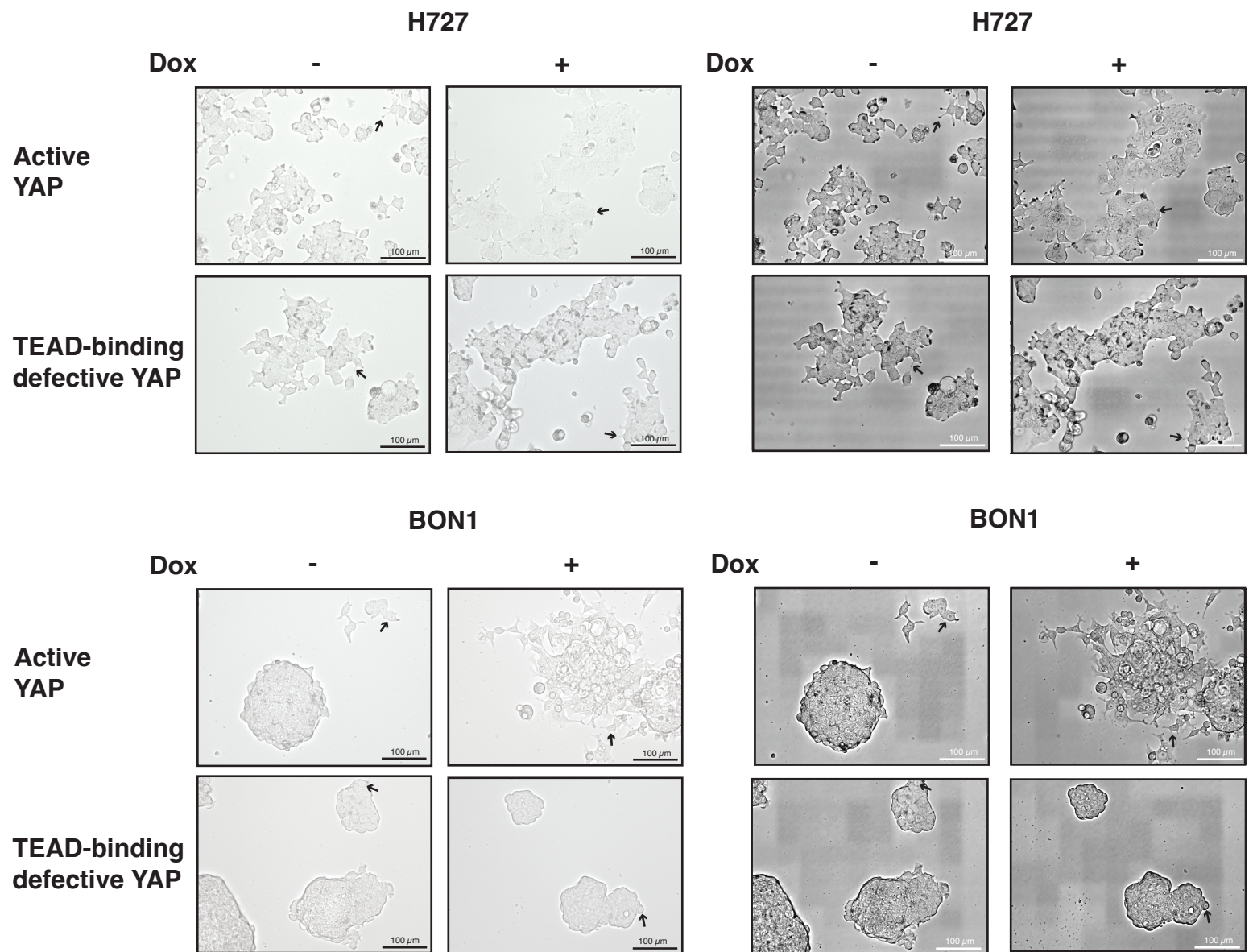

Supplement: Supplemental Figure S1 — Disrupted YAP-TEAD binding inhibits neuroendocrine morphology in carcinoid cells. Bright-field microscopy at ×200 total magnification for control (−Dox), active YAP, or TEAD-binding defective YAP overexpression (+Dox) cells. When active YAP was overexpressed in H727, cells lost their thin dendritic-like cell processes (arrows). Similarly, BON1 cells lost their rounded shape (arrows). Cell images were deblurred using the Cellpose deblurring algorithm and presented alongside original images. Scale bar: 100 μm. Original magnification: ×200. Dox, doxycycline. [file mmc7.pdf]
