# Supplementary material for: YAP and TEAD Are Transcriptional Regulators of Neuroendocrine Differentiation and Growth in Carcinoid Cells
Source: Am J Pathol. 2025 Nov 20;196(2):345–58. doi: 10.1016/j.ajpath.2025.10.012 (PMC12881680; doi:10.1016/j.ajpath.2025.10.012)
Supplement: Supplemental Figure S2 — YAP and TEAD chromatin immunoprecipitation sequencing (ChIP-seq) based on k-means clustering. Cluster-specific heatmaps. ChIP-seq signal represents fold change of YAP (A) or TEAD (B) immunoprecipitation (IP) sequencing reads relative to input chromatin. n = 2 YAP sequencing reads; n = 2 TEAD sequencing reads. [file mmc8.pdf]

Supplementary Figure 2

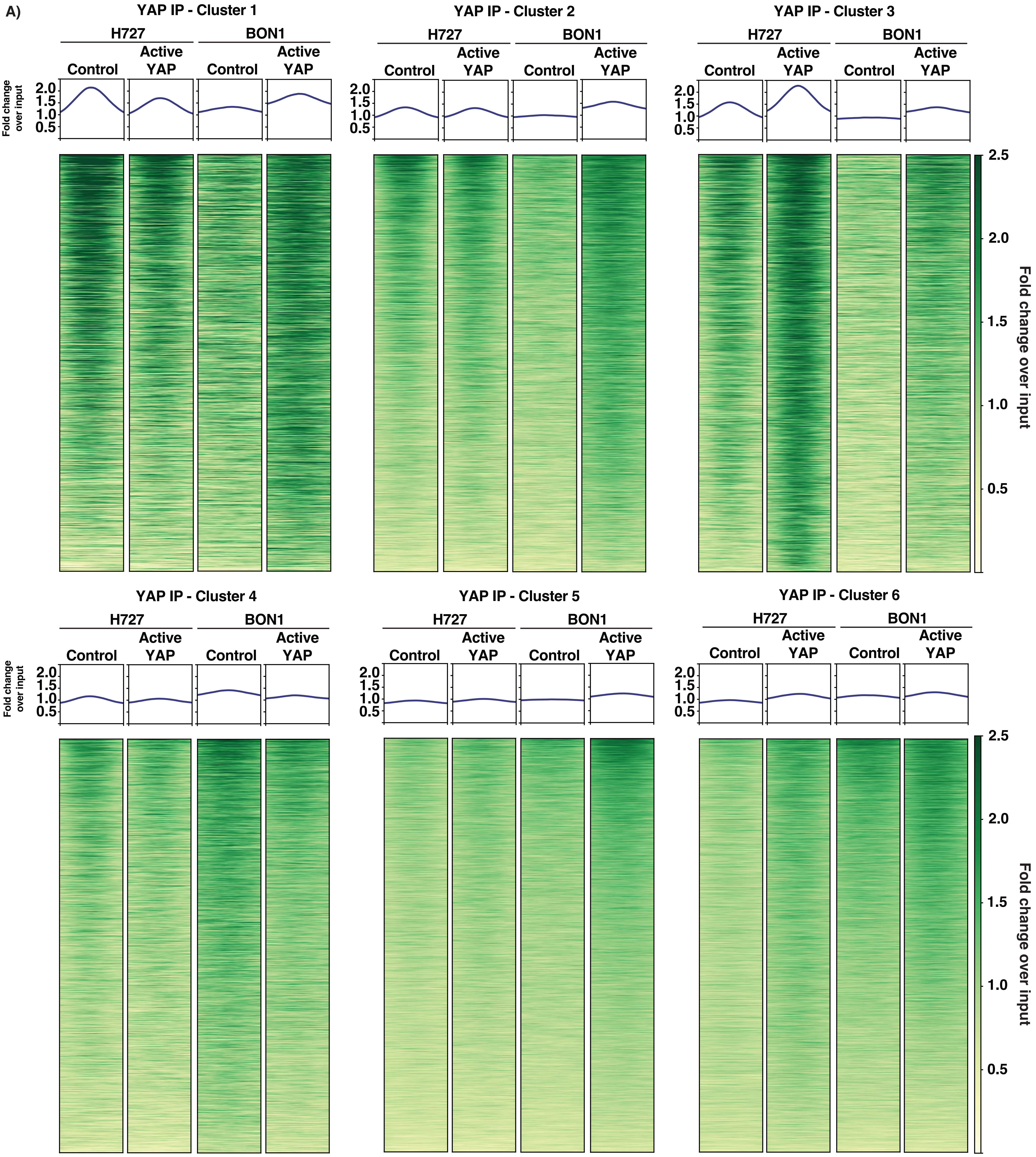

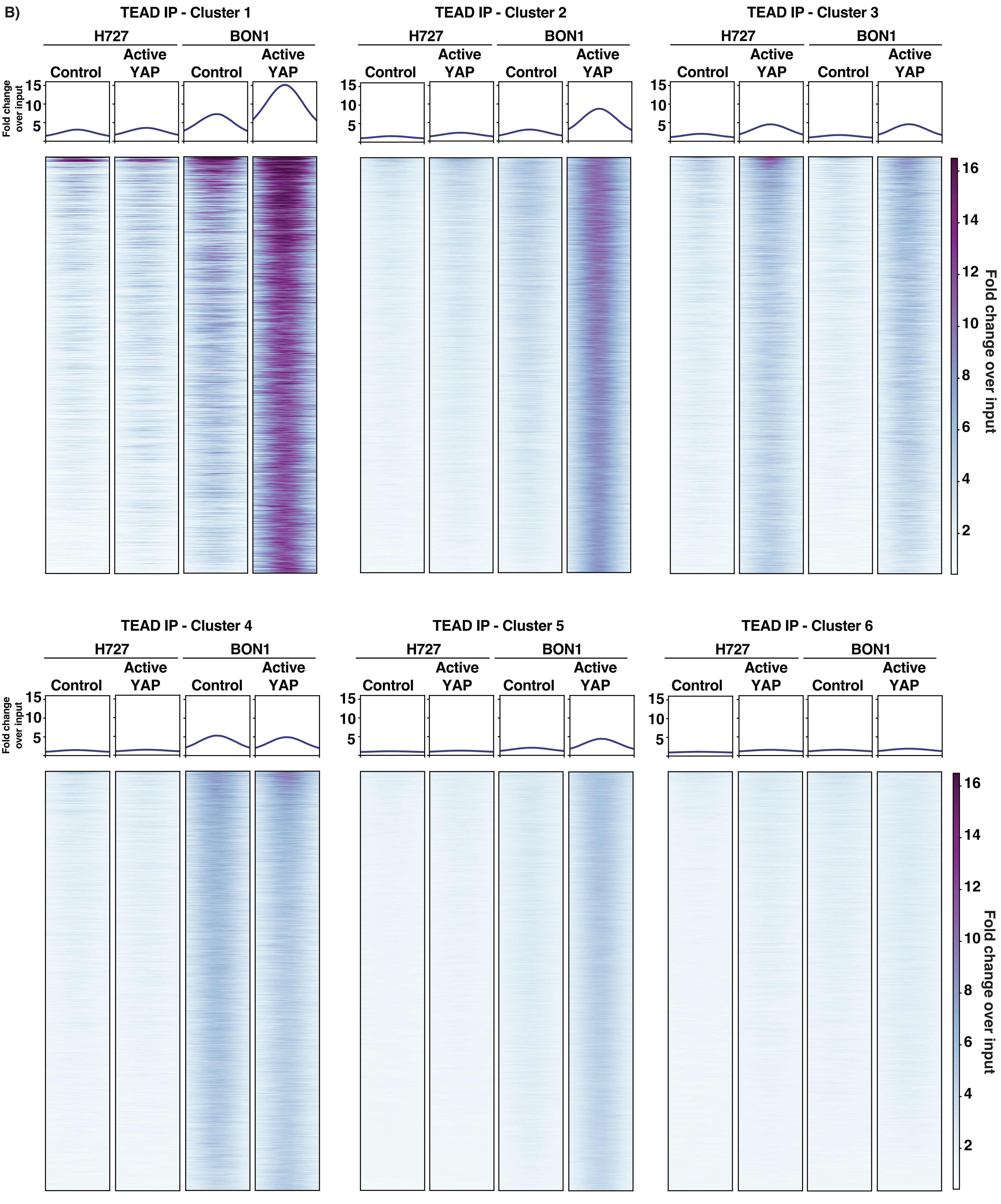
B)
TEAD IP - Cluster 1
TEAD IP - Cluster 2
TEAD IP - Cluster 3
TEAD IP - Cluster 4
TEAD IP - Cluster 5
TEAD IP - Cluster 6
H727
BON1
Control
Active YAP
Fold change over input
15
10
5
16
14
12
10
8
6
4
2
Fold change over input
