# Supplementary material for: YAP and TEAD Are Transcriptional Regulators of Neuroendocrine Differentiation and Growth in Carcinoid Cells
Source: Am J Pathol. 2025 Nov 20;196(2):345–58. doi: 10.1016/j.ajpath.2025.10.012 (PMC12881680; doi:10.1016/j.ajpath.2025.10.012)
Supplement: Supplemental Figure S3 — Integrative genome view of ASCL1, INSM1, NEUROD1, and NKX2-2. Chromatin immunoprecipitation (ChIP) peaks (YAP and TEAD DNA-binding sites) were identified within promoters and proximal enhancers of BMP4, TGFBR2, and JAG1 or within 1 Mb of the transcription start site (TSS) of ASCL1, INSM1, NEUROD1, and NKX2-2. Predicted promoter and enhancer regions were obtained from ENCODE. ChIP-seq tracks were displayed for control and active YAP overexpression conditions in H727 and BON1, with YAP or TEAD immunoprecipitation (IP). [file mmc9.pdf]

Supplementary Figure 3

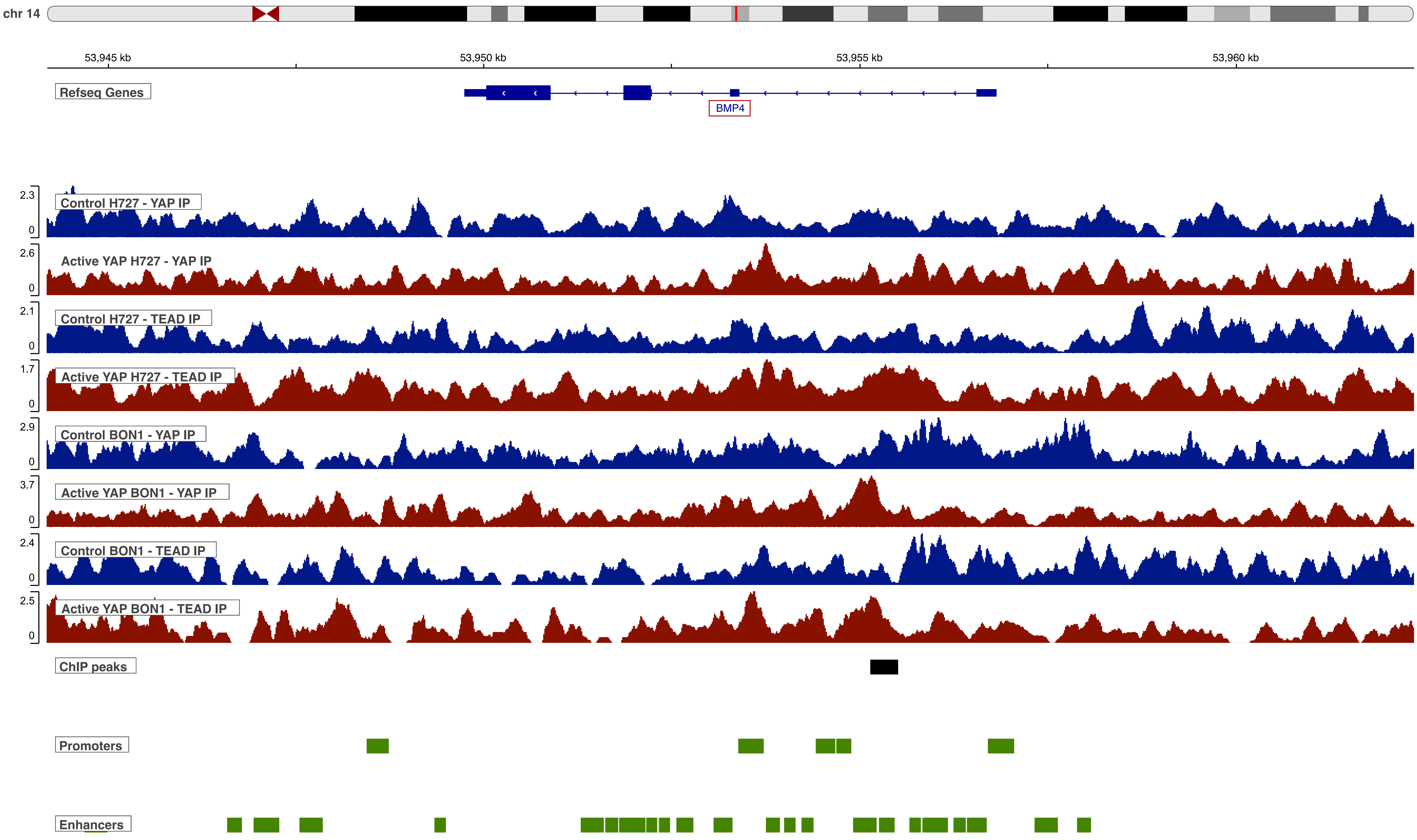

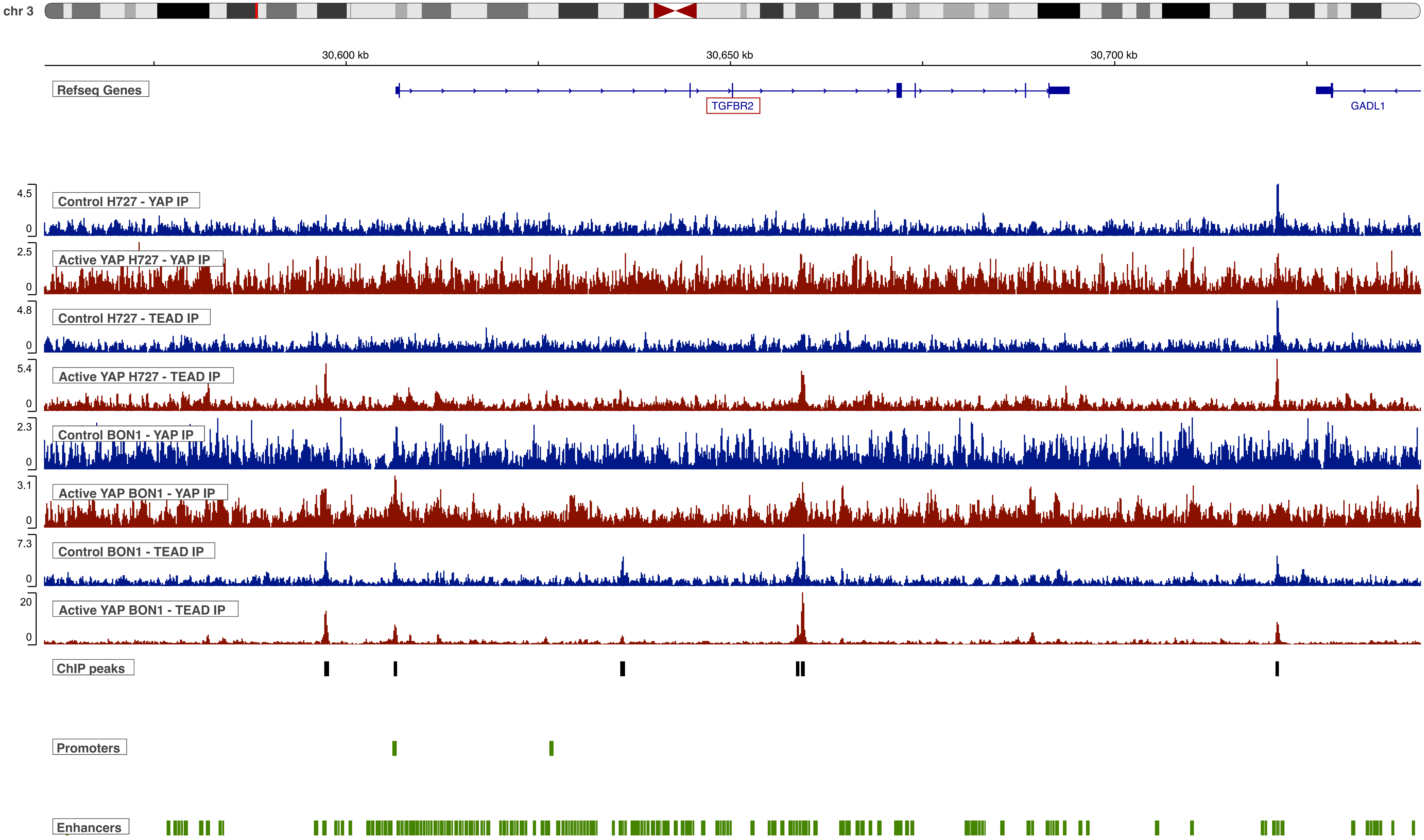

chr 3
30,600 kb
30,650 kb
30,700 kb
Refseq Genes
TGFBR2
GADL1
4.5
0
Control H727 - YAP IP
2.5
0
Active YAP H727 - YAP IP
4.8
0
Control H727 - TEAD IP
5.4
0
Active YAP H727 - TEAD IP
2.3
0
Control BON1 - YAP IP
3.1
0
Active YAP BON1 - YAP IP
7.3
0
Control BON1 - TEAD IP
20
0
Active YAP BON1 - TEAD IP
ChIP peaks
Promoters
Enhancers

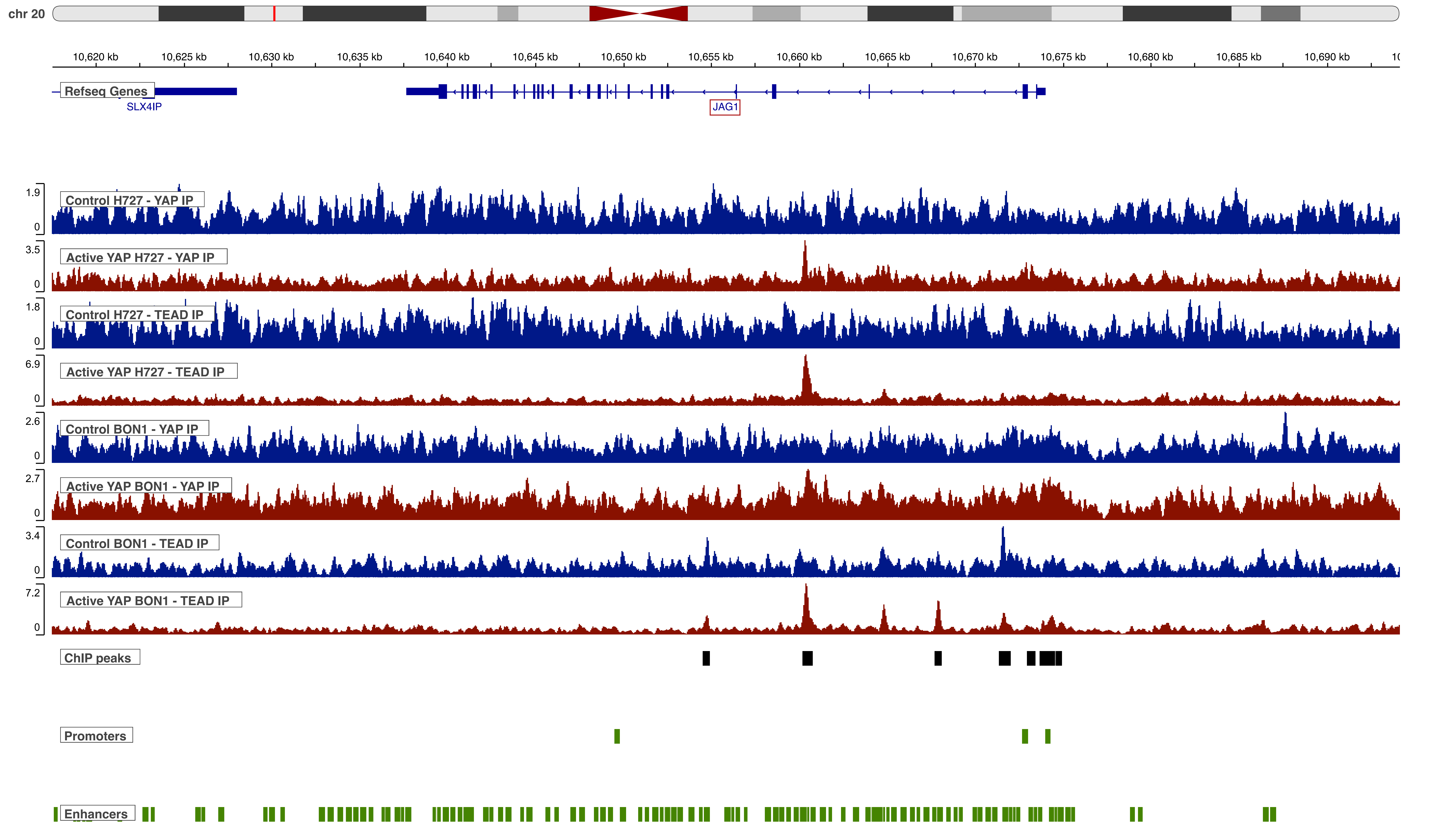
chr 20
10,620 kb
10,625 kb
10,630 kb
10,635 kb
10,640 kb
10,645 kb
10,650 kb
10,655 kb
10,660 kb
10,665 kb
10,670 kb
10,675 kb
10,680 kb
10,685 kb
10,690 kb
Refseq Genes
SLX4IP
JAG1
1.9
0
Control H727 - YAP IP
3.5
0
Active YAP H727 - YAP IP
1.8
0
Control H727 - TEAD IP
6.9
0
Active YAP H727 - TEAD IP
2.6
0
Control BON1 - YAP IP
2.7
0
Active YAP BON1 - YAP IP
3.4
0
Control BON1 - TEAD IP
7.2
0
Active YAP BON1 - TEAD IP
ChIP peaks
Promoters
Enhancers

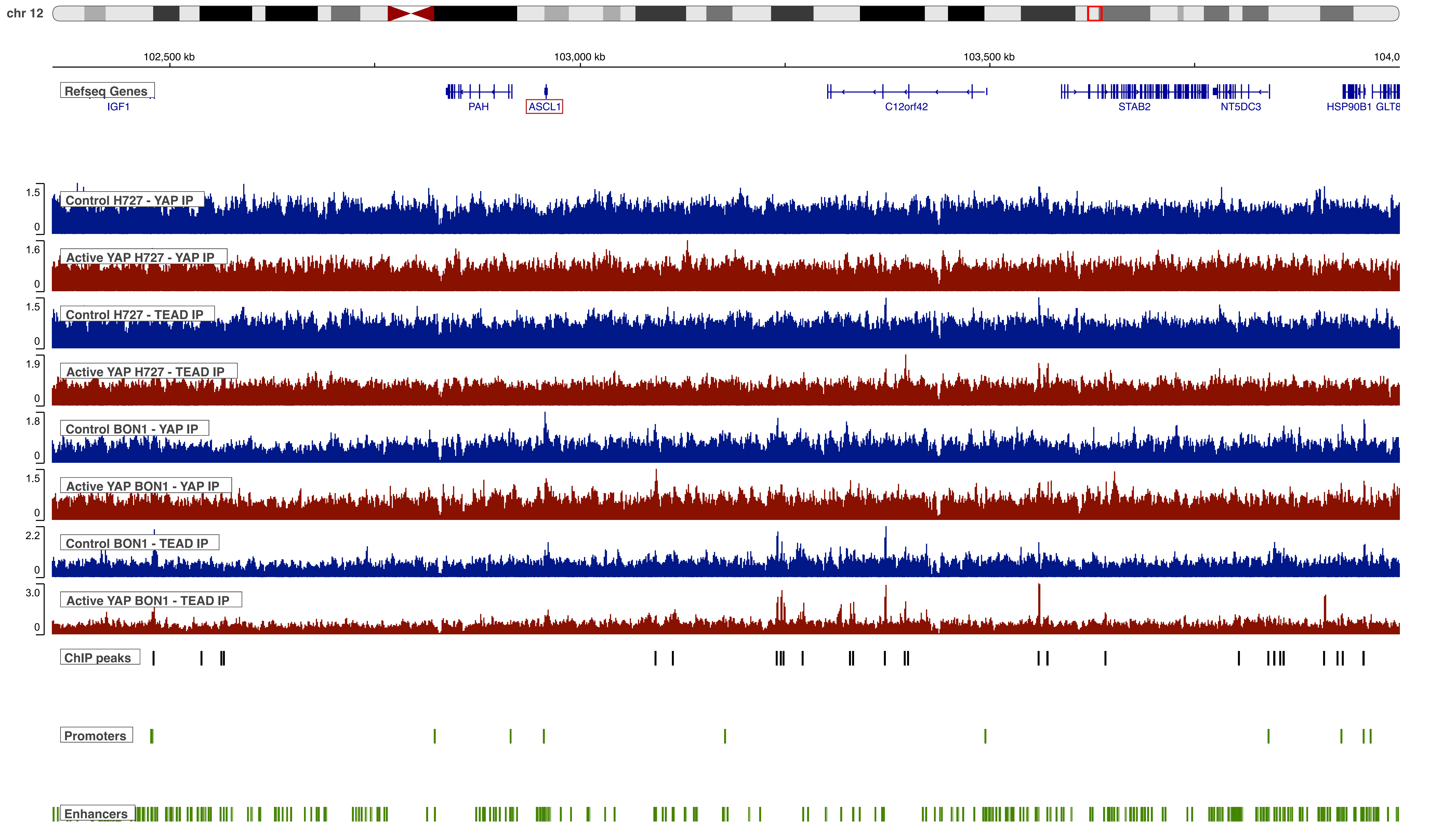

chr 12
102,500 kb
103,000 kb
103,500 kb
104,0
Refseq Genes
IGF1
PAH
ASCL1
C12orf42
STAB2
NT5DC3
HSP90B1 GLT8
1.5
0
Control H727 - YAP IP
1.6
0
Active YAP H727 - YAP IP
1.5
0
Control H727 - TEAD IP
1.9
0
Active YAP H727 - TEAD IP
1.8
0
Control BON1 - YAP IP
1.5
0
Active YAP BON1 - YAP IP
2.2
0
Control BON1 - TEAD IP
3.0
0
Active YAP BON1 - TEAD IP
ChIP peaks
Promoters
Enhancers

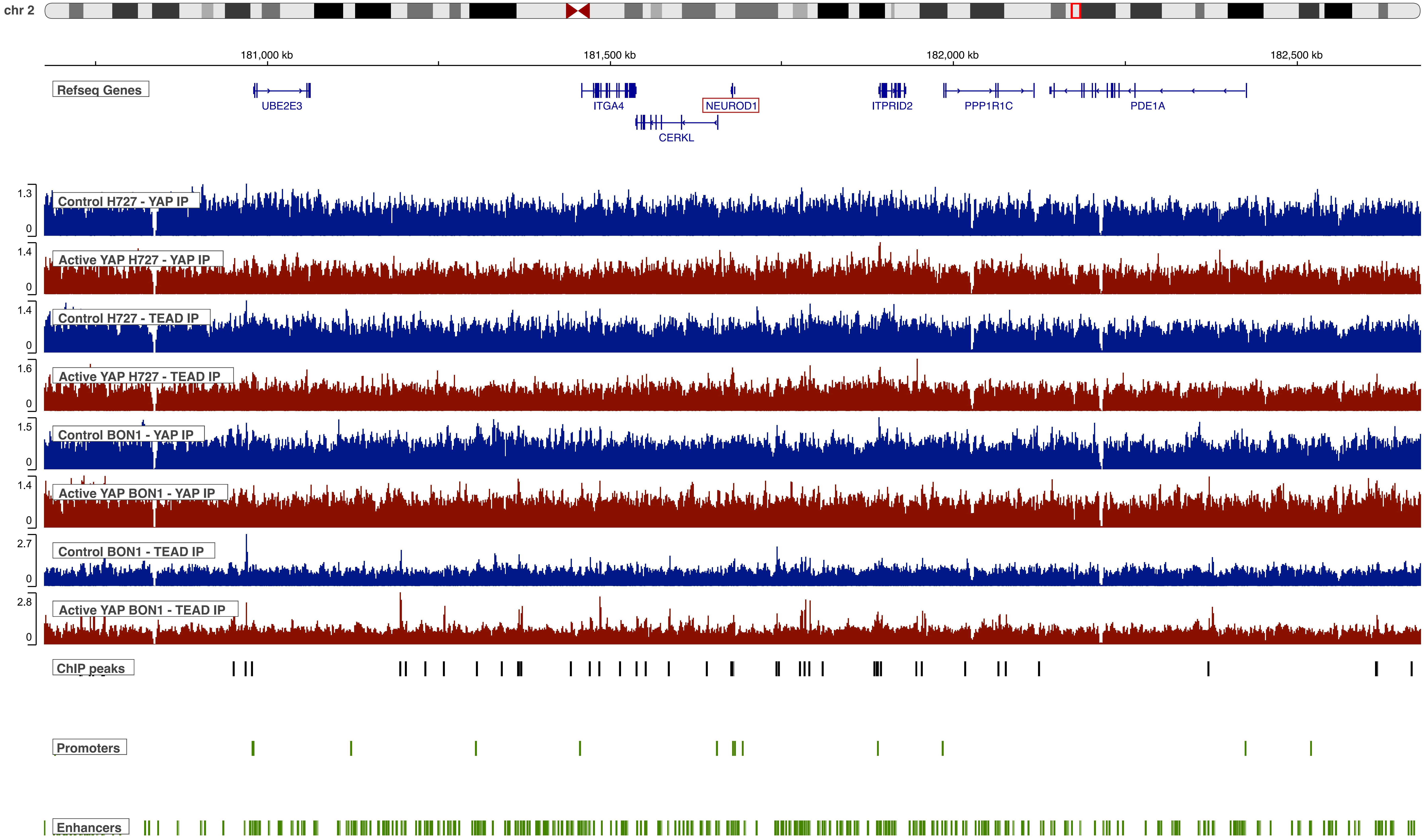
chr 2
181,000 kb
181,500 kb
182,000 kb
182,500 kb
Refseq Genes
UBE2E3
ITGA4
NEUROD1
CERKL
ITPRID2
PPP1R1C
PDE1A
1.3
0
Control H727 - YAP IP
1.4
0
Active YAP H727 - YAP IP
1.4
0
Control H727 - TEAD IP
1.6
0
Active YAP H727 - TEAD IP
1.5
0
Control BON1 - YAP IP
1.4
0
Active YAP BON1 - YAP IP
2.7
0
Control BON1 - TEAD IP
2.8
0
Active YAP BON1 - TEAD IP
ChIP peaks
Promoters
Enhancers

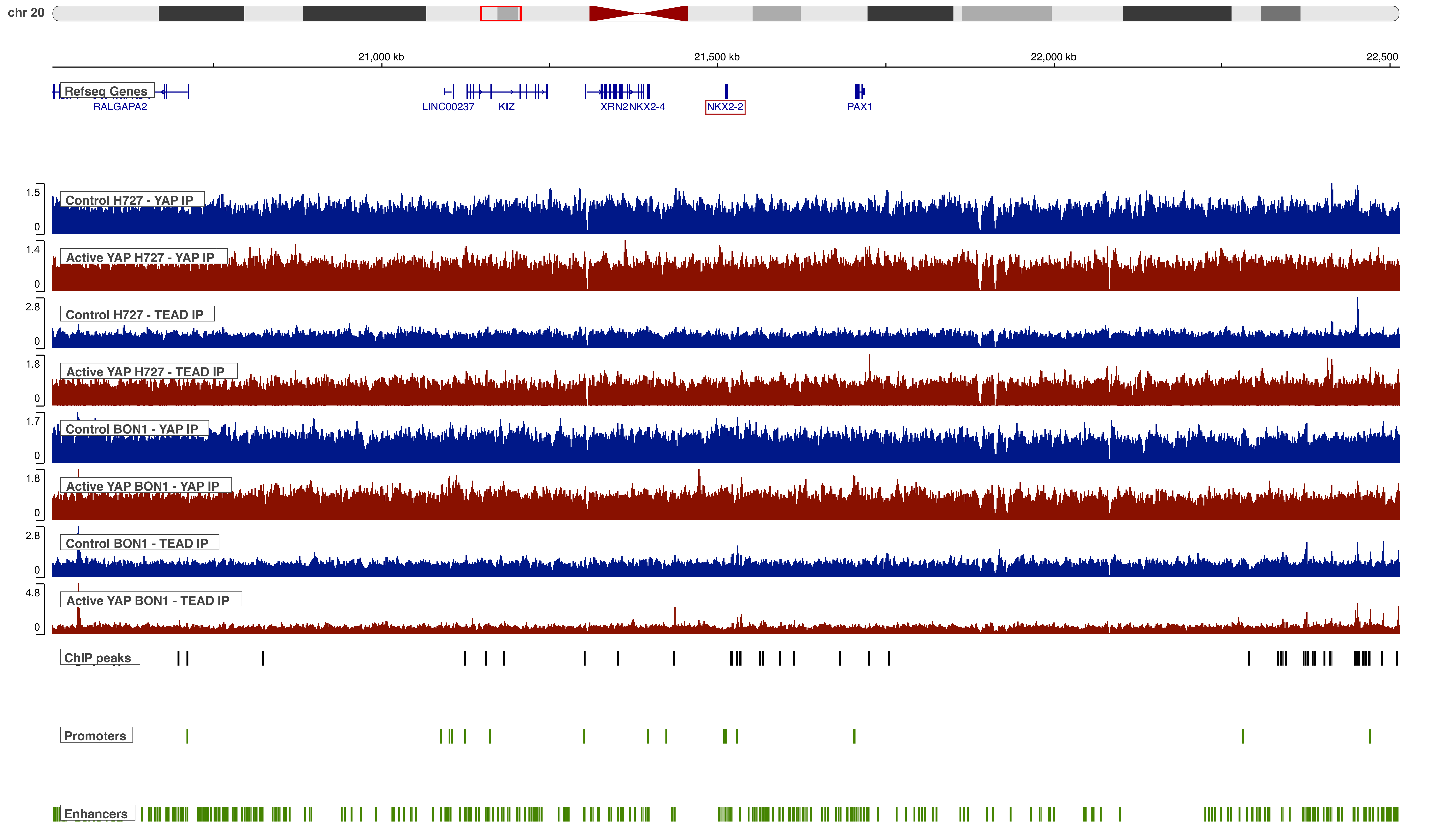
chr 20
21,000 kb
21,500 kb
22,000 kb
22,500
Refseq Genes
RALGAPA2
LINC00237
KIZ
XRN2
NKX2-4
NKX2-2
PAX1
Control H727 - YAP IP
Active YAP H727 - YAP IP
Control H727 - TEAD IP
Active YAP H727 - TEAD IP
Control BON1 - YAP IP
Active YAP BON1 - YAP IP
Control BON1 - TEAD IP
Active YAP BON1 - TEAD IP
ChIP peaks
Promoters
Enhancers

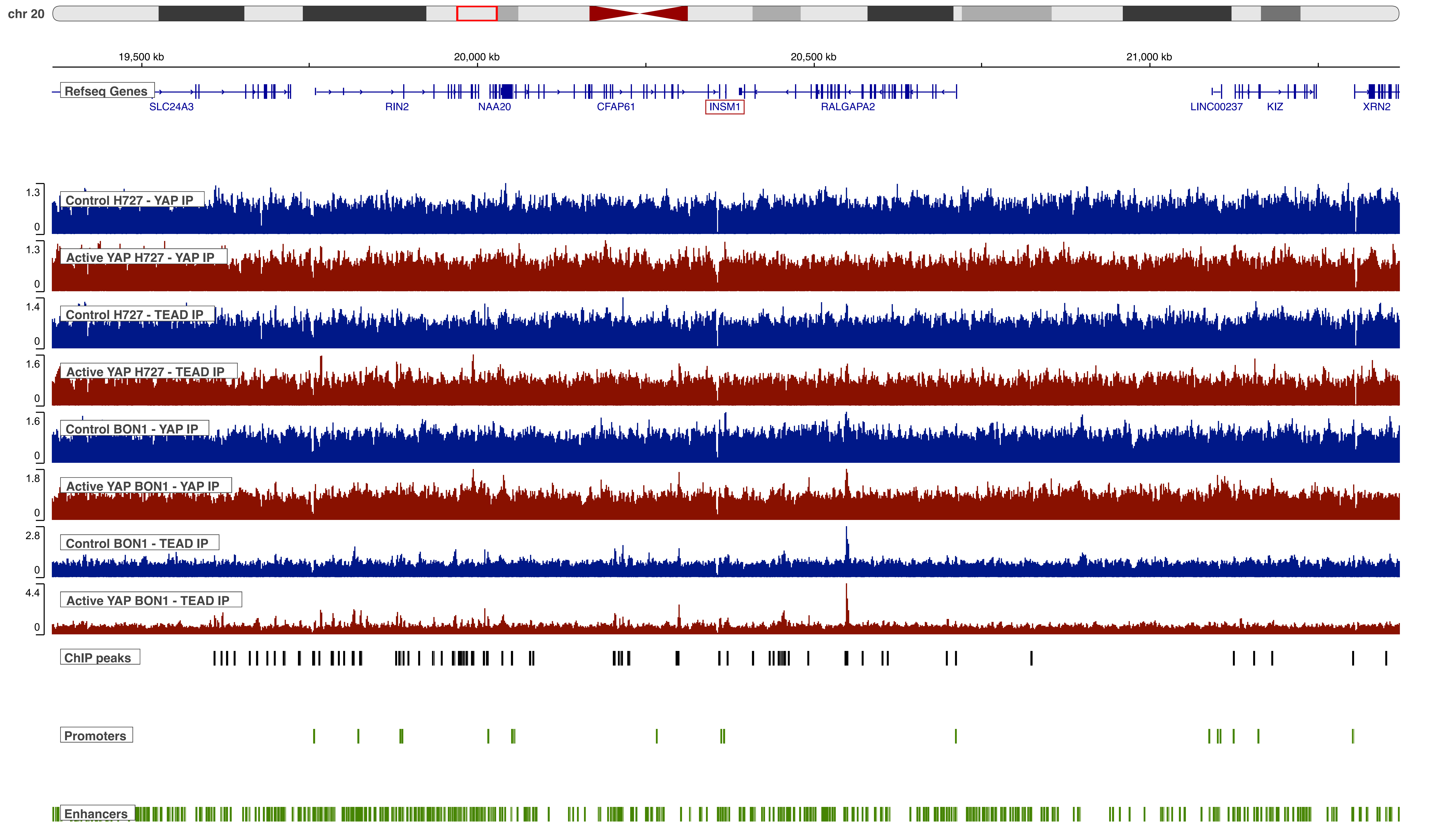

chr 20
19,500 kb
20,000 kb
20,500 kb
21,000 kb
Refseq Genes
SLC24A3
RIN2
NAA20
CFAP61
INSM1
RALGAPA2
LINC00237
KIZ
XRN2
Control H727 - YAP IP
Active YAP H727 - YAP IP
Control H727 - TEAD IP
Active YAP H727 - TEAD IP
Control BON1 - YAP IP
Active YAP BON1 - YAP IP
Control BON1 - TEAD IP
Active YAP BON1 - TEAD IP
ChIP peaks
Promoters
Enhancers
